# Optimizing Performative Skills in Social Interaction: Insights From Embodied Cognition, Music Education, and Sport Psychology

**DOI:** 10.3389/fpsyg.2019.01542

**Published:** 2019-07-16

**Authors:** Andrea Schiavio, Vincent Gesbert, Mark Reybrouck, Denis Hauw, Richard Parncutt

**Affiliations:** ^1^Centre for Systematic Musicology, University of Graz, Graz, Austria; ^2^Faculty of Social and Political Sciences, Institute of Sport Sciences, Université de Lausanne, Lausanne, Switzerland; ^3^Musicology Research Unit, KU Leuven, Leuven, Belgium; ^4^Department of Musicology, IPEM, Ghent University, Ghent, Belgium

**Keywords:** embodied cognition, interaction, skill acquisition, music education, sport psychology

## Abstract

Embodied approaches to cognition conceive of mental life as emerging from the ongoing relationship between neural and extra-neural resources. The latter include, first and foremost, our entire body, but also the activity patterns enacted within a contingent milieu, cultural norms, social factors, and the features of the environment that can be used to enhance our cognitive capacities (e.g., tools, devices, etc.). Recent work in music education and sport psychology has applied general principles of embodiment to a number of social contexts relevant to their respective fields. In particular, both disciplines have contributed fascinating perspectives to our understanding of how skills are acquired and developed in groups; how musicians, athletes, teachers, and coaches experience their interactions; and how empathy and social action participate in shaping effective performance. In this paper, we aim to provide additional grounding for this research by comparing and further developing original themes emerging from this cross-disciplinary literature and empirical works on how performative skills are acquired and optimized. In doing so, our discussion will focus on: (1) the feeling of being together, as meaningfully enacted in collective musical and sport events; (2) the capacity to skillfully adapt to the contextual demands arising from the social environment; and (3) the development of distributed forms of bodily memory. These categories will be discussed from the perspective of embodied cognitive science and with regard to their relevance for music education and sport psychology. It is argued that because they play a key role in the acquisition and development of relevant skills, they can offer important tools to help teachers and coaches develop novel strategies to enhance learning and foster new conceptual and practical research in the domains of music and sport.

## Introduction

Expert musicians and skilled athletes often display the stunning ability to adapt to, and coherently engage with, the shifting demands of their contingent milieu. A sudden change in the tempo of a music performance or the emergence of a particular spatial configuration of players in team sports requires the immediate generation of appropriate novel actions to keep the music “alive” or the sport performance possible. Traditionally, this process is described as a largely automatic mechanism, where little or no attention is dedicated to the generation and enactment of the new actions (see Dreyfus and Dreyfus, 1986; Schmidt and Wrisberg, 2008). These forms of *skillful coping* are fluidly integrated within one’s repertoire of action, such that no explicit thought is necessary for them to be implemented (see Dreyfus, 2002). Indeed, according to this standpoint, there is no need for reflection at this stage because our cognitive systems can detect and select the most adequate behavioral outcomes in response to the unfolding contingencies of a given context. It has been argued that the *automaticity* of such mechanisms develops through a progressive shift from an initial phase where skills are acquired to a final performative stage where the task (e.g., repeating and elaborating an “error” to make it sound intentional in improvised music or dribbling the opponent in ball games) can be achieved without any explicit “cognitive” involvement (c.f., Papineau, 2013). By this view, musicians and athletes do not follow pre-defined rules as they become experts; it is only at the beginning of the process, when skills are acquired and developed, that these schemas need to be examined and discussed. Therefore, a novice singer will explicitly consider whether her\his posture and breathing technique are consistent with what she\he has learnt from her\his teacher before starting a performance, just as the beginner basketball player will be paying attention just before taking the shot to whether her\his shooting style aligns with her\his coach’s guidelines. But when expertise has been acquired and one is fully “absorbed” in the dynamics of the event, so the story goes, there is no time for inferential mediations − i.e., an awareness of the kinematics of a given action is arguably no longer necessary (e.g., Araújo and Davids, 2011). A soccer player does not have to think about his or her movements when dribbling an opponent, just like the expert rock guitarist does not have to explicitly recall each position of the fingers during a solo performance (see Menin and Schiavio, 2012). “Conceptualizations” and “explicit reflections” are part of the process only “when the agent’s absorption fades away or is not yet ready. In this perspective, [conscious] representation comes either before skill acquisition or after skilful performance, i.e., it is either a temporary scaffold necessary to automatize a routine (as during training), or a conceptual expedient to rationalize its defining principles a-posteriori” (Cappuccio, 2015, p. 219).

Positions based on similar insights have contributed an important perspective to the understanding of expertise and performance. However, they also entail some necessary limitations, particularly when the entire process of acquiring and optimizing skills is considered to be a march toward thoughtless automaticity. First, the strict dichotomy between *conscious processes* and *mindless behavior* may appear too static to capture the complexity of the phenomenon. As Sutton and colleagues comment:

“because practitioners in many skilled movement domains know that self-conscious thought can disrupt well-practised actions, they like to entrust grooved action sequences to the body, to the habitual routines of kinaesthetic memory. But because they also know that open-ended, flexible performance is context-sensitive and, in the ideal, exquisitely responsive to subtle changes in a situation, they also want to be able to bring all of their experience to bear in the moment, to bring memory and movement together, with thought and action cooperating instead of competing” (Sutton et al., 2011, p. 80).

Second, the entire process of skill acquisition is often conceived of as an individual achievement.

Many of the contributions reported in Table 1, for example, appear to remain neutral on whether individual skills should be regarded as inherently social and on the role that social aspects may play in their acquisition and development. However, if we look at the concrete settings where skills are acquired, we find that researchers, educators, and coaches are increasingly considering the importance of participation and reciprocal interaction^1^.

**Table 1:** Examples of previous research on skill acquisition.

Author(s)	Major findings/claims
Miller, 1956; Gobet et al., 2001	Skill acquisition is moving from processing and executing component task units at the bottom level toward Gestalt processing at the top level: grouping information into chunks
Fitts and Posner, 1967	Skill acquisition involves three stages: cognitive, associative, and autonomous
Cundey, 1978	Skill is any competent, rapid and accurate performance, including a wide range of mental activities
Newell and Rosenbloom, 1981	The effect of practice time on performance for cognitive and perceptual-motor skills
Adams, 1987	Skill forms a wide domain of possible behaviors; skill must be learned; skills are defined by motor performance in attainment of a task-specific goal
Holding, 1989	All human skills involve the coordination of perception and action
Schmidt, 1993	Skill acquisition is based on information processing and selection of adequate motor programs
Dreyfus, 1996	Skill acquisition transforms our relation to the world
VanLehn, 1996	Different kinds of skills involve cognitive or intellectual ability: from explicit, slow, detached processing to implicit, automatic, and engaged performance
Ericsson and Lehmann, 1996	Expertise is maximal adaptation of the performer to the task-environment
Hurley, 1998	Expert performance carries out actions or processes that are intentional but not consciously intended
Ingold, 2001	Combined anthropological and ecological approach to skill
Rosenbaum et al., 2001	Skill is the ability to achieve goals within some domain with the increased likelihood as a result of practice
Ericsson, 2006, 2008	Expert performance is related to active engagement in deliberate practice. Major role of immediate feedback, problem-solving, and evaluation, and opportunities for repeated performance

Recent approaches in music education, sport psychology, and motor control place a strong emphasis on the inherently *social contingencies* of performative activity in sports and the arts (see e.g., Borgo, 2005; Davids et al., 2007; Hauw, 2018; Schiavio et al., 2018a,b). Here the conscious process of “explicit rule-following” leading to tacit knowledge has been traded for more nuanced, fluid, and flexible understandings of how optimal skills are developed (Bril, 2002; Araújo and Davids, 2011). This move is particularly useful for reconsidering the role of internal models based on mental representations. In musical contexts, for example, mental representations of desired performative outcomes are traditionally thought to offer an excellent scaffolding to assist the students’ learning trajectory (Lehmann, 1997; Gruhn, 2006; Hallam and Bautista, 2018). Such representations are arguably developed through hours of “studying the score and playing the music” (Lehmann and Jørgensen, 2018, p. 134), being shaped by a rich variety of self-monitoring and evaluation strategies (e.g., McPhail, 2013). In other words, this model portrays musical learning as an input–output process mediated by the internal cognitive laws of the individual. This way, the mismatch between the predicted outcome (understood in terms of a mental representation) and the actual performance becomes the main focus of the training, with its minimization being the general goal of learning.

In more recent years, young musicians and performers are increasingly encouraged to freely explore their musical potential and the best way to express themselves in interaction with others, avoiding the constant self-monitoring of their actions (see Bowman, 2004; Borgo, 2007; Schiavio and van der Schyff, 2018). Similarly, recent accounts in sport psychology have emphasized the adaptive nature of athlete-environment interactions during practice and performance (e.g., Hristovski et al., 2012; Seifert et al., 2014). With regard to this point, it has been shown that participants in team sports can collaboratively modify their offensive and defensive strategies to co-adapt to changing environmental constraints (e.g., the opposite team) (see Duarte et al., 2012). Such adaptability has been described in music and sport at both *behavioral* and *phenomenological*^2^ levels (e.g., Schiavio and Høffding, 2015; Hauw, 2018; Rochat et al., in press), with demonstrations of how self and other, behavior and experience, are in fact intrinsically interdependent at various levels and timescales. Jacobs and Michaels (2006), in a similar vein, considered expert performance as a dynamical system comprising performer, tools, the environment, and other individuals. Rodger (2010, unpublished) also argues that skill acquisition involves the development of tight action-perception couplings, leading to the recruitment of both bodily and environmental resources.

In this article, we aim to provide additional grounding for this line of research by comparing and further developing original themes concerning how performative skills are acquired and optimized by novices in the context of music and sport. In particular, we will draw on recent research in *embodied cognitive science* (ECS) with the aim of developing a more integrated view of how action and thought shape each other dynamically. Scholars inspired by ECS emphasize the deep continuity between perception, action, biological organization, reflection, and intersubjectivity (Di Paolo et al., 2017). By this view, talking about the physical location of the mind becomes meaningless. Instead, “mind” is here conceived as an emerging property of the interplay between a brain–body system and the contingent (social, cultural, physical) environment in which the organism is situated (Thompson, 2007). Drawing from these insights, we argue that because musicians and athletes often learn in groups and share experiences, actions, cultures, and “histories of structural couplings^3^” with their surrounding world (Varela et al., 1991), ECS offers important conceptual resources to capture the rich web of contextual contingencies that music and sport entail. Our claim is that embodied and phenomenological insights can help reconcile thought and action, as well as individuality and collectivity − which are often conceived of as separate when looking at skill acquisition.

The paper is structured as follows: in the next section, we introduce the main tenets of ECS, focusing on its phenomenological and interactive groundings. In line with recent work by Gallagher (2017), Chemero (2009), and Fuchs (2017), it is argued that explanations of the mental cannot be limited to the individual’s brain–body system (e.g., his or her internal biological norms), nor should they entail a separation between high-level and low-level cognitive processes. Instead, we maintain that mind depends on the dynamical interplay of brains, bodies, and the social, cultural, and physical features of the environment (Clark, 1997, 2006; Donald, 2001; Malafouris, 2013). Focussing on the role of intersubjectivity, we then consider how social contingencies and reciprocal interactions can offer novel possibilities to acquire, develop, and optimize performative skills. In doing so, we examine (1) the feeling of being together, (2) the capacity to skillfully adapt to the contextual demands of the social environment, and (3) the development of distributed forms of bodily memory. In conclusion, we discuss how these categories are relevant to future research, theory, and the practical issues related to how music teachers and sport coaches can develop novel strategies to enhance and optimize skill acquisition.

## Reconciling Dichotomies

### An Open Mind

ECS offers a non-reductive view of mental life − one that brings together insights from disciplines such as theoretical biology, linguistics, phenomenology, aesthetics, constructivism, ecological psychology, and complex systems theory, among others (see e.g., Shusterman, 2009; Stewart et al., 2010; Colombetti, 2014). In its broadest sense, the central idea of ECS is that physical resources from the entire body of a living system and its environment participate in driving cognitive processes. Therefore, our capacity to think, feel, reason, and interact with others depends directly on the ongoing patterns of interaction between a brain–body system and its niche (Johnson, 2007; Clark, 2008). Because factors external to the brain are said to co-constitute the mind, this approach offers a useful alternative to more traditional accounts of mentality, which are often based on an individualist and internalist perspective.

To illustrate the point, we might consider the following two approaches: *functionalism* and what we define as *internalist embodiment*. While different on many levels, both approaches share a common assumption − namely, that cognition is a property of the individual. Functionalist psychologists identify cognition with information-processing operating in the individual’s head, metaphorically equating minds with computer devices (see Fodor, 1983). To understand a psychological state, according to this framework one should not focus on its physical make-up; what really matters, instead, is the functional role it plays for the cognitive economy of the system. This move, it is argued, allows researchers to better explore the complexity of mental phenomena and create specifications general enough to capture a wide variability in physical implementation^4^. However, the kinds of generalizations necessary to explain cognitive life do not go beyond the system where the psychological state is individuated. Psychology, so to speak, remains intrinsic to the system that displays the causal properties required to instantiate the mental state being studied.

As an example of “internalist embodiment” instead, let us now briefly consider the work on body-formatted (or B-formatted) representations (see Goldman and de Vignemont, 2009). The central idea here, as explained by Gallagher (2017), pp. 4–5, is that the brain can develop internal representations with a specific bodily related content, without them being propositional – or conceptual – in format. Because the content of these representations may involve interoceptive states (e.g., physiological states, visceral sensations, etc.), motor goals (e.g., the control and monitoring of behavioral outcomes), and social contingencies (e.g., the understanding of the intentions of another person, realized by the perceiver’s mirror-like activity^5^), they are thought to play a good explanatory role in ECS. However, some argue that the appeal to internal models based on representations still fails to capture the unity of action and perception, thought and action, and subjectivity and intersubjectivity. In fact, on this view “social cognition […] is embodied only to the extent that B-formatted representations involved in perceptual mirroring are used to represent the actions or mental states of others” (Gallagher, 2017, pp. 4–5). In both functionalism and internalist embodiment, the mind is thus described in terms of the living system’s *internal* factors. Functionalists look for the causal role each mental state plays for the system’s operational functioning, while defenders of the other account would consider the integration of bodily and neural states of the agent as constitutive of mentality. For both, however, the external world remains, in a sense, *detached* from the internal processes that truly instantiate mental activity. Internal and external resources are *discontinuous* with each other − they are part of separate domains.

More recent scholarship inspired by ECS and phenomenological philosophy offers a different view. If we are to consider the body as a constitutive tool for cognition, we cannot but examine the body in its dynamical interplay with its environment. The body, in other words, does not operate in a vacuum (Chemero, 2009). Because of this, ECS emphasizes the necessarily full involvement of body and world for the realization of mental life. This involves patterns of behavioral, emotional, and social adaptivity that are enacted within a contingent milieu, giving rise to a complex *brain–body-environment system*, where aspects inherent to each of them are mutually relevant for its maintenance and development (Varela et al., 1991). Such a view resonates with earlier insights on the notion of “functional system” discussed by Luria (1966), who defined *flexibility* as the set of constant and coherent goals implemented by the responses emerging from the environment. However, ECS does not conceive of the relationship between the living system and environment as captured by a stimulus-response schema. Instead, ECS scholars often argue that there is a mutual adaptation between niches and living systems that constantly shifts the trajectories of inner and outer constraints, making the recourse to inputs and outputs superfluous. These scholars therefore increasingly draw from the resources offered by *dynamical systems theory*−a mathematical tool adopted to explore how complex systems develop in time through the convergence and divergence of its elements (Strogatz, 1994; Kelso, 1995). Work inspired by such insights trades the adoption of stimulus and response for sets of “differential equations that express the magnitude of variability between pairs of (non-linearly) coupled components” (van der Schyff et al., 2018), applying it to a vast range of domains, including music (Large et al., 2016; see also Schiavio et al., 2017a; van der Schyff and Schiavio, 2017a,b; Walton et al., 2014).

Because this unfolding network of interactivities defines an open horizon of viable opportunities for action, ECS can help us describe the flexible processes whereby skills are acquired and developed (Schiavio et al., 2017b). In particular, as ECS gives equal importance to structures and processes internal and external to the living system, an approach to skill acquisition informed by such a view assumes strong *continuity* between the intrinsic biological organization of living systems, their phenomenology, and their capacity to generate and maintain stable relationships with the environment^6^ (Weber and Varela, 2002).

### Collective Dynamics and the Emergence of Optimal Skills

Consider two young amateur musicians improvising music together, or two non-professional athletes participating in a pick-up basketball game. As they perform together, expertise and social understandings are developed collaboratively, requiring mental and behavioral resources to be fluidly integrated. While it is easy to see how existing skills can be improved through participation in such practices, it is more complicated to explain how novel skills might emerge. Indeed, here knowledge is not transmitted between the two (it is assumed that the participants have similar expertise), nor can it be detected in the environment (no instructions are given by a teacher or coach).

A first solution might involve what is usually defined as “folk psychology”−each agent interprets what the other is doing in terms of his or her own mental states (e.g., intentions, desires, drives, etc.). By systematically analyzing and predicting each other’s minds, novel behavioral configurations can emerge as adaptations to what the other is (about to be) doing. According to this interpretation, it could be stated that interactors constantly monitor and modulate their existing motor patterns in light of what others might do, giving rise to modifications in their actions, which then give rise to novel skills. At a closer look, however, this view implies that the achievement of a given goal (e.g., playing a vibrato at the right time during an improvisation, finding the open player during a fast-break in basketball) still remains based on the stored sensorimotor repertoire that is available to the individual agent (see Proctor and Vu, 2006). Indeed, following the classic description of James (1890), action plans are selected by a cognizer in terms of their immediate consequences. This, however, entails a possible paradox. How can performance in joint situations become effective if the motor possibilities relevant for the immediate contextual contingencies are limited by the vocabulary of actions accessible to the singular individual? Consider how a classical violinist might respond to an unexpected crescendo by the pianist with whom she\he is playing. Are already existing *internal* resources manipulated and transformed in light of the specific contextual demands? Or does the generation of new valuable behavioral strategies require the living system to negotiate in real time between both *internal* and *external* resources?

To answer these questions, it may be helpful to discuss examples of improvised and collective music-making activities (Borgo, 2007; Heble and Laver, 2016) and private practices and deliberate play sequences in sports (Côté et al., 2007; Laurin-Landry, 2018; Uehara et al., 2018).

Children and adolescents often spend their recreation time at school in activities such as backyard versions of soccer or street-style basketball. In these situations, they are free to experiment with various movements and interactions and explore their physical possibilities. Because the rules of these pick-up games^7^ are flexible and often apposite to the context, they can both adapt to these rules and make the rules adapt to them. Such “deliberate play” therefore provides relevant opportunities to improvise, explore, learn, and negotiate contextually novel behavioral solutions. For instance, in order to take part in a street soccer game with older − and more skilled − opponents, a child must develop novel repertoires of action (e.g., technical and tactical skills) that are consistent with the kinds of possibilities that can be explored and that will equip him or her to keep participating in the unfolding dynamics of the match. Reading the opponent’s mind is not enough. Adaptation has to be fast, fluid, dynamic, and contextually meaningful. Similarly, when improvising with an expert jazz musician, one might discover that differences in expertise are bigger than expected. However, novel solutions to optimize the performance might emerge through moment-to-moment interactions: both musicians can adapt to each other − e.g., a solo can be intentionally repetitive to enable the other to explore novel harmonic possibilities and progressions. In the context of music education, it has recently been argued that settings where performance and collaboration are prioritized may foster important benefits in terms of negotiating differences and stimulating trust and social understandings (Higgins and Mantie, 2013), leading researchers to focus on informal learning practices (Green, 2001, 2008). In these contexts, (musical) meanings are recursively and collaboratively transformed, giving rise to creative outcomes that do not involve prescriptive rules to be followed or mind-reading mechanisms (see van der Schyff et al., 2016). Similarly, Ryan and Schiavio (2019), among others, advanced the hypothesis that music-making is inherently *extended*, suggesting how cultural, social, and physical resources (internal and external to the agent) are fluidly integrated and constitutive of the performance outcome. By this view, even categories like “agency” can become distributed across individuals, giving rise to a complex network of collective experiences that can contribute to individual practice.

In sport psychology, recent research has offered similar insights. Adopting qualitative methodologies based on interviews that enable athletes to re-enact their previous experiences, several studies have focused on the collaborative dynamics in various sport settings. Examples include a match in table tennis (Sève et al., 2002), a competitive exercise in acrobatic sports (Hauw and Durand, 2004), and the attempt to finish an ultra-trail running race (Rochat et al., 2017, 2018). These studies emphasize the strong recursive interplay between bodies-in-action, internal states, and extrabodily resources^8^. It might therefore be argued that athletes’ actions are properties emerging from the fluid integration of internal and external components (see also Semin and Cacioppo, 2008; Hwang et al., 2018; van Opstal et al., 2018). Importantly, such elements can only be exploited through action, giving rise to continuous loops where athletes shape and are shaped by the various contextual contingencies associated with (the goals of) each performance. Here the structural unity between behavioral and phenomenological processes has been addressed in cases where agents are able to monitor and supervise “from within” the dynamics of their own performance^9^.

The wide range of examples provided here shows how new possibilities for action can be developed and negotiated in real time as a performance unfolds. Unidirectional forms of learning are then traded for more dynamical “explorations” of the different possibilities for action emerging from the interactions and from the affordances of the environment (see Schiavio and Cummins, 2015; Schiavio, 2016). Here, it appears that “forms of flexible and adaptive actions which are clearly not the product of deliberation or explicit reflection can nonetheless be best understood as involving certain sorts of (dynamic, embodied) intelligence” (Sutton et al., 2011, p. 78). The experience of performing with one or more other individuals cannot be reduced to a simple “mindless” response to an external perturbation. It emerges from, and sustains, the ongoing interactive coupling; it brings together emotional, bodily, and cultural aspects that may not be present in individual contexts; it is continuous with a wide range of unique metabolic and neural processes; and gives multiple agents shared responsibilities (e.g., the maintenance of the interaction) among others. As the joint activity unfolds, different behavioral trajectories are developed, with a shared horizon of meaningful possibilities for novel (inter)actions being co-created. This embodied dynamicity helps eschew the dichotomy between behavioral and reflective domains and, at the same time, shifts the unit of analysis from the individual to the group. Here, what is meaningful becomes what is *shared.* On this view, skills are “relational” in the sense that they are shared and negotiated by a community of practice, and developed and experienced contextually. The move can be particularly useful to differentiate intersubjective experiences from cases where living systems engage with the physical tools of the environment (e.g., a musical instrument, a ball, etc.). While both dimensions are important for the acquisition of optimal skills, each has its own phenomenology and core principles – as such, they might be best described autonomously. In what follows, we focus on social, embodied, experience. In doing so, we first individuate three categories that can arguably only emerge in such context and then explore their role in the participatory foundations of skill acquisition.

## Skills Beyond the Individual

Many individual skills in music and sport are often optimized and developed collaboratively, through an (inter)active effort based on reciprocal adaptation and phenomenological awareness (see Montero, 2010). In many cases, performance and learning largely overlap, integrating self and other, action and perception, and doing and knowing in the contextual dynamics of action. To better understand how this could be so, this section examines three main dimensions of skill acquisition: (1) the feeling of being together, as meaningfully enacted in collective musical and sport events; (2) the capacity to skillfully adapt to the contextual demands from the social environment; and (3) the development of distributed forms of bodily memory.

### The Feeling of Being Together

The patterns of coordinated behavior occurring between sport teammates or members of a music ensemble are not fully preestablished; instead, they are constantly being shaped by the environmental contingencies that agents learn to master and exploit during performance (Fuchs and De Jaegher, 2009; Di Paolo et al., 2010; Araújo and Bourbousson, 2016; Schiavio and van der Schyff, 2016). Gesbert and Durny (2017), for example, highlighted how the objectives pursued by soccer players in competition (i.e., trying to score a goal while maintaining the team’s defensive balance) are constantly open to interpretative processes that emerge from their interactions with the environment. Depending on the musical score, the feedback from the audience, the moment in the match, the teammate with the ball, the positioning of opponents, the area where the ball is situated, and so on, each musician or player can constantly reinterpret the unfolding situation in ways that are meaningful for the whole ensemble or team. The pursued objectives of individual players, in a way, are thus never fully “individual” − they are developed, transformed, and manipulated in light of specific collective constraints, opening a new horizon of possibilities for joint action. Along these lines, other studies have shown how a specific sensitivity to environmental information enabled co-performers and team members to grasp the state of the group’s coordination through the feeling of being together − or not being together − with others (see Lund et al., 2012, 2014). A significant part of these experiences corresponds to the immediate feelings of being affected by others (Colombetti and Torrance, 2009; He and Ravn, 2017). According to Himberg et al. (2018), the feeling of acting with others is an essential part of collective performance: because in certain situations, the activity of individuals depends on reciprocal interaction with others, one is required to actively take part in participatory processes of skillful co-adaptation. This involves shared forms of emotionality, intelligence, and coordination − action and perception of co-performers coexist in a continuous coupling where individual experience is constituted in part by what constitutes the other’s experience (Froese and Di Paolo, 2011; Tanaka, 2017).

A similar scenario was discussed in a recent study by Schiavio et al. (2018a); see also Gande and Kruse-Weber, 2017. Here, a program for informal pedagogy was examined by means of qualitative interviews collected with “facilitators” − expert musicians who guided the sessions and enabled the participants to discover musical possibilities through collective improvisation and coordinated music-making. The novices who participated in the program were asked to reciprocally interact and adapt to the specific demands of each session (e.g., drums, choir, etc.). The study shows how the categories of collaboration, non-verbal communication, and sense of togetherness were continuous with more general cognitive processes related to meaning-making, and fostered a shared sense of community through musical (inter)action. The feeling of being together, particularly, allowed the novices to generate music organically − as a whole. The program, from the perspective of the “facilitators”, brought out a sense of being a group, which allowed the attendees to help each other and actively seek musical configurations that were appropriate for their cultural backgrounds and their ongoing experience.

In the context of sports, Lund et al. (2012) described how rowers gradually learned and developed the experience of a joint rhythm by becoming increasingly attuned to their mutual interaction. The tension felt between their movements during performance allowed them to mutually adjust their activity, suggesting that co-performance is realized through an on-line integration of internal and external resources, where awareness and meanings are developed through interactive forms of action (see also Schaffert et al., 2011, for a focus on how auditory feedback can facilitate the adjustments of performance in rowing). In the same vein, Gesbert et al. (2017) described how soccer teammates during no-possession phases of the ball were attuned to the recognition of their expected defensive configuration before shifting to the collective work of ball recovery. The perception of such expected configuration − and of the associated feeling of being together − was crucial for each player for starting this collective work. Here, the movements of only one player, because of his position or speed of replacement, could simultaneously prevent the recognition of this collective configuration and the perception of being together. As coordination is constantly re-played by the dynamics of individual activities, the feeling of being together is constantly under threat. Mutual sensitivity to these fluctuations in experience is therefore considered as one of the main characteristics of collective expertise (see e.g., Saury et al., 2010). Gesbert and Durny (2017), moreover, described how two players sharing the same objective during a soccer counterattack (quickly attacking the opposing goal) were able to develop highly specific expectations on how to attack the opponent goal. As they drew closer to the opposing goal, one of them (Phil) became sensitive to the quick decrease in the distance that separated his ball-carrying teammate (Andrew) from the opponent (situated in front of him), understood that he did not have the same expectations as Andrew, and sought to adjust his behavior. While this example clearly showed how the experience of soccer players is distributed across different layers of individual and collective awareness, it should also be noted that individuals display various degrees of sensitivity to such an experience. Indeed, in the same case, Andrew was focused on other environmental information and did not perceive the tenuousness of this feeling of being together. He was not sensitive to the slowing down of Phil’s ball call and did not adapt his activity to this information.

### Skillful Adaptation

While being attuned to the feeling of being together, teammates often make use of and (re)interpret environmental information to skillfully adapt to the needs of collective behavior (Walton et al., 2015; Bourbousson and Fortes-Bourbousson, 2016). This helps co-performers engage with a vast range of contingencies in meaningful ways, developing patterns of action and perception that are constitutively dependent on their mutually adaptive behavior (see Fuchs and De Jaegher, 2009). With regard to this point, one can consider three adaptation modalities that have recently been observed *via* inductive analysis in soccer-specific situations: these are called “local,” “global,” and “mixed” (Gesbert and Hauw, 2017).

The “local” mode describes an adaptation of the player’s activity to his proximal social environment, such as the behavior of a nearby opponent or a teammate ball-carrier and one’s direct opponent (e.g., moving away from the direct opponent to offer a pass solution to the partner carrying the ball). The “global” mode describes the adjustment of an activity to the collective organization of a part of the team. The “mixed” mode, finally, accounts for adaptations to the activity of a nearby opponent or partner and those of more distant agents. A recent study (Gesbert et al., 2017) focused on these modalities by studying how soccer team players mutually adapt to each other during competition. As team coordination emerged, many players achieved good sensitivity to it by constantly shifting between these modalities. Similarly, work in progress in synchronized swimming (Gesbert and Hauw, in preparation) aims to characterize the interaction modalities that these swimmers use in order to predict how they will interact as they cope with different environmental perturbations. Adopting a phenomenologically inspired method based on qualitative interviews, the authors describe how two swimmers (Monica and Isabella) managed to organize their behaviors and adapt to each other despite substantial difficulties in maintaining optimal distance^10^. During a training session, their coach prompted them to actively seek each other, stimulating a reorganization of existing patterns of behavior on the basis of their capacity to adapt to each other. The coach did not ask them to manipulate their existing motor knowledge; instead, he modified the task constraints (e.g., their distance) to promote a novel set of actions that required a sensitive *reconfiguration* of their consolidated motor knowledge, which then led to the acquisition of novel skills and expertise. Again, resources that emerge when agents meaningfully interact are seen to play a constitutive role in developing individual skills (Reed and Bril, 1996; Hutto et al., 2015). This aligns with data from a recent qualitative study comparing musical learning experiences in individual and collective settings (Schiavio et al., 2019). Here it was found that while students often rely on their teacher to optimize their performative skills, they can also benefit from an active interaction with their peers: by exchanging musical ideas, providing feedback to each other, improvising together, and sharing their personal experiences, novices find new possibilities to transform and develop their technical, expressive, and communicative, musical skills. The benefits of joint learning are also recognized by music teachers, when they purposely “step back” to leave their students with more freedom and responsibilities for their own learning (Schiavio et al., 2018a). This suggests that the capacity to skillfully adapt to the contextual demands of the social environment is not only present in experts, but can also be found in novices – albeit in a more learning-oriented sense.

In expert athletes, finally, Lund et al. (2014) observed improved synchronization of professional trampolinists’ movements when the athletes remained engaged in the process of jumping together^11^. Here the authors describe how the athletes became progressively sensitive to their partners’ jumps through the sound produced by the two trampolines during training^12^. In other words, adjustment modalities can be developed and enhanced through a different, cross-modal, interaction process − athletes directly feel and shape the other’s performance, and learning opportunities are continuously renegotiated by synchronizing with others. Put simply, it seems that without interaction, new behavioral solutions appropriate for this context might not emerge.

### Distributed Bodily Memory

According to Fuchs (2017), p. 341, distributed bodily memory involves “an ensemble of behavioral and interactive dispositions characterizing the members of a social group that have developed in the course of earlier shared experiences and now prefigure similar interactions of the group”. Consider the classic work by Sudnow (1978), where a detailed phenomenological description of the learning trajectory necessary to develop adequate improvisatory skills on the piano is put forward. While the main focus of Sudnow remains the author’s own body and his subjective experience, the role of the *other bodies* is not dismissed. In fact, in an oft-cited passage of the book, Sudnow describes the intense feeling of watching his mentor Jimmy Rowles performing on stage as follows:

“I watched him night after night, watched him move from chord to chord with a broadly swaying participation of his shoulders and entire torso, watched him delineate waves of movement, some broadly encircling, others subdividing the broadly undulating strokes with finer rotational movements, so that as his arm reached out to get from one chord to another it was as if some spot on his back, for example, circumscribed a small circle at the same time, as if at the very slow tempos this was a way a steadiness to the beat was sustained” (Sudnow, 1978, p. 82).

Such occurrence, Sudnow admits, played an important role in helping him develop the ability to improvise and produce similar musical phrases on the piano. Through the body of his mentor, he gained access to new motor possibilities and configurations that he then explored autonomously. In this case, individual behaviors were forged by a shared experience, and developed after the event to be further elaborated in other situations. In other cases, however, co-actors might have no time to wait, and must reciprocally interact and share their skills as the performance unfolds. Consider the following example reported in an ongoing study with a team of eight synchronized swimmers (Gesbert and Hauw, in preparation). During choreography, one of the swimmers (Barbara) managed to lead the other swimmers without having the opportunity to perceive them visually. How was she able to do this? She explains:

“At this moment, I know that the swimmers are a little too far apart after the last movement, so I’m doing a breaststroke of a certain length, I’m sensitive to the amount of movement in the water so they can follow me and we’re all at the right distance.”

Interestingly, when Barbara was outside the pool, she was unable to describe where she had to stop. She only verbalized her experience when she was able to move into the pool. Her movements affected what she sensed, allowing her to gain access to “mental landscapes,” words, and experiences that were not present before. When she felt she had reached the *right length*, she decided to stop. While this decision emerged intuitively from her embodied activity in the pool, her behavior was clearly under the influence of all the past situations, which constrained it (see Sutton, 2007; Fuchs, 2012, 2016; Sutton and Williamson, 2014). Said differently, the enaction of this *specific feeling about length* was linked to the breaststroke patterns that she had built from earlier experiences through multiple interactions with other swimmers in this specific configuration. In a sense, therefore, the patterns of action sedimented in Barbara’s bodily memory do not really belong to her, nor are they to be considered a property of her individual activity. They can be actualized through her lived body, but they have been developed through the embodied interaction with other swimmers (see Hauw and Bilard, 2017). In this view, Barbara, her teammates, the pool, the coach, and their patterns of interactivity have all become a constitutive part of her learning trajectory, with different dynamical configurations individually enacted as part of a collective process. Barbara’s swimming style, in other words, can be understood as a continuous adaptation to the patterns of reciprocal interaction involving people and things. To better capture this idea, we may refer here to the notion of “degeneracy” (Bernstein, 1967; Mason, 2010; Kelso, 2012; Komar et al., 2015). Degeneracy is a term to describe how the same outcome can be achieved in many ways using different components. For instance, it has been shown that expert basketball players may use lower and upper body joints (and limb segments) differently to shoot successfully while coping with various task constraints, including the distance to the basket or the position of the nearest defenders (Davids et al., 2013). In other words, degeneracy accounts for the various possibilities in which athletes adapt their behaviors to the interacting social or physical constraints of the performative environment they are embedded in.

In a recent paper, Gesbert and Durny (2017) described how soccer players can regain ball possession by exploiting specific and independent goals. According to their position on the pitch (forward, axial midfielder, side-offensive midfielder, etc.), they each developed a specific and compatible mutual understanding of what was going on. Even though they were attuned to common environmental information (such as their own defensive style and/or the opponent’s offensive configuration to recognize a potential situation of regaining ball possession), they were no longer deliberately sensitive to the activity of their teammates. After regaining ball possession, the players directly foresaw what would happen both individually and collectively. They “saw” things before they happened through their capacity to re-enact their previous contextual experience and project it against the new contextual demands. The rehearsal of these realistic situations in training brought about fundamental changes in the way the players perceived their environment. In fact, the development of this bodily memory enabled them to unburden their attentional resources, facilitating that performance. At that moment, all the interactive dispositions that the soccer players had developed through repeated interactions in past competition and/or training were intuitively re-enacted. Through collective bodily memory, each player’s movements were correctly understood and put into context by his teammates just before regaining ball possession, improving their capacity to predict and adapt their behavior (e.g., “we are not in position …Wilson is a bit too far from his opponent…So I do not engage in pressing”). Last, as noted earlier, Lund et al. (2012) described how two rowers (one was a novice) learnt to coordinate rhythm. Their results highlighted how the experience of mutual synchronization mediated by coupled ergometers enabled them to develop a kinaesthetic, implicit memory of poor rhythm and gave them the possibility of monitoring their own performance (Sutton and Williamson, 2014). The rowers’ interlaced movements sometimes involved divergent experiential qualities described by the novice rower as a “tjuk-tjuk” – or the feeling of *not being together* (see section “The Feeling of Being Together”). For instance, as she sometimes had the tendency to slide in the seat, she noted how this movement was linked to the experience of a poor rhythm characterized as movements that work against each other. This feeling of “tjuk-tjuk” became an immediate kinaesthetic reminder for the rowers during performance, helping them re-establish the correct rhythm.

## Conclusion

Because skills are often understood as properties of single agents, their acquisition is conceived of as an individual process based on internal dispositions, talent, and individual practice. However, frameworks based on solitary achievements may lead to the assumption of a strong discontinuity between inner psychological states and external behavior. A focus on automatic responses (i.e., “mindless” action) may not resolve this dichotomy. Indeed, positing strict automaticity in response to a given environmental perturbation in order to explain music-making or sport performance would keep high-level and low-level processes separate. As Sutton et al. (2011), p. 89, insist, “theorists tend to evacuate psychology entirely from action, running the risk of thus neglecting the complex interplay between embodied dynamical factors and cognitive factors”. As we have seen, however, ECS offers a way forward: by shifting the unit of analysis from individual behavior to the collective dynamics, we can better comprehend how collaboration contributes to learning skills, how adaptations to the performative event are often enacted in socially meaningful environments, and how habits and repertoires of actions are distributed within the relevant community of practice.

These insights might call for an important reconceptualization of learning settings. Tools to understand and develop skills inspired by ECS may help teachers and coaches find novel ways to enhance the learning process. For example, numerous studies in music and sport have shown how learners use *preferential behaviors* (e.g., Lund et al., 2012; Laroche and Kaddouch, 2015) – that is, they spontaneously re-enact particular configurations that previously led to an optimal performance. Coaches and teachers can thus help learners explore areas of practice that fall outside their spontaneous achievement zone in various collective situations. This process requires a familiarization with the interaction dynamics involving (1) new opportunities based on the feeling of being together, (2) constant adaptation to the environmental resources being explored, and (3) an awareness of the distributed forms of bodily memory to help performers take decisions together and act as one when needed. There should be points of continuity across areas, and exploration might be developed in autonomy or under constant supervision. In both ways, each learner can flourish and push herself to reach novel achievements from a well-known starting point and by reciprocally relying on the interactor. By exploring novel opportunities together, learners can be “in-the-moment” together, with the possibility to also share thoughts and impressions on their practice.

A promising way to take this dimension into account in learning settings involves the explicit use of “re-enactment” techniques. In the sport context, for example, videos are extensively used to provide post-performance feedback and help the athletes to (re)connect the feelings and outcomes of their activity (e.g., Hauw, 2018). However, the feedback is very often associated with normative remarks on the outcomes or behavior (i.e., “you should do it like this!”). For this reason, it has been argued that a stronger effect might be obtained by integrating videos with a form of reflective practice (e.g., Hauw, 2009). Here, the aim is not to compare a performance to a model of expected or ideal behavior, but instead to generate a re-enactment – or a form of artificial re-living of the experience − with the support of the video, which can then be linked to a normative example in order to work on it. Hauw (2018) described how the use of such re-enactments was able to solve problems or resolve disruptions in motor behavior, to modify a counterproductive involvement in competition, and to supervise athletes’ long-term development. In these cases, athletes are asked to relive the feelings (including body sensations) that they have experienced in various situations. This involves an effort to “disconnect” from the present moment and project oneself into a previous situation. The athletes are able to describe how they managed their interactions with the environment and how they created their own situations (Hauw and Durand, 2007; Villemain and Hauw, 2014; Mohamed et al., 2015; Antonini Philippe et al., 2016; Gesbert et al., 2017; Gesbert and Durny, 2017; Rochat et al., 2018). Videos or other traces of past activity are thus very useful for maintaining the markers that preserve the dynamics of an activity. And indeed, it should be noted that re-enactment techniques are not limited to visual information only, but can also involve auditory signals (Pizzera et al., 2017; see also Sors et al., 2015, and Schaffert et al., 2019 for reviews). In general, these interventions can help learners to again “feel” the dynamics of action without any overt movement. *Prima facie*, it seems that the kind of exploratory behaviors associated with the three categories described above are here lost. Instead, there are ways to stimulate a sort of mental exploration: athletes can be offered the opportunity to analyze their own activity by assessing the relevance of their actions for the context or they can be prompted to consider new possibilities by provoking a shift in the way they experience their activity. For example, are there other more efficient ways to reach high performances? How can collective activities between team members be promoted and developed in future interactions? Athletes can also be simultaneously prompted to evaluate their performance with regard to their individual and collective adaptive possibilities (e.g., MacNamara and Collins, 2011; Gesbert et al., 2018). In this last case, discussions and shared reflection would still provide the feeling of being together and thus potentially elicit possibilities for adaptation that are developed collectively. Similar practices can thus help capture the non-dichotomous nature of thought and action, providing important methodological insights that can be useful for understanding the main properties of skill acquisition and future developments in education and pedagogy. In music education, a similar account might involve the adoption of so-called “conscious strategies” and the recourse to metacognitive competence (see Concina, 2019). As reported by Nielsen (1999), Jorgensen (1995) argues that with problems about learning goals, the contents of the piece, the available learning media, time allocation, and methods needing to be explicitly considered and addressed. A possible extension of such practices might include the “mental exploration” (Høffding and Schiavio, 2019) of collective situations, where individual agents are asked to explicitly account for interactions and joint situations. While there is no agreement on how to coherently implement similar practices across different contexts, the adoption of such strategies may be an apt counterpoint to improvisational music pedagogies and less formalized forms of sport and play.

## Data Availability

All datasets analyzed for this study are included in the manuscript and the supplementary files.

## Author Contributions

AS made major contributions to content in all areas of the paper. VG and DH made substantial contributions to sections “Skills Beyond the Individual” and “Conclusion.” MR and RP provided comments and suggestions throughout the development of the paper that were implemented in the final draft. All authors approved the final version of the paper.

### Conflict of Interest Statement

The authors declare that the research was conducted in the absence of any commercial or financial relationships that could be construed as a potential conflict of interest.

## Footnotes

^1^Because of their performative dimensions, sport and music are two ideal domains to explore the social, embodied, and creative dynamics involved in skill acquisition. While they both display different traditions, contexts, and specific objectives, one point of continuity between these areas precisely involves the monitoring of one’s skills, as well as their development and optimization.

^2^Phenomenology is a philosophical tradition that explores the structures of experience and consciousness, associated with authors like Husserl, Merleau-Ponty, and Heidegger.

^3^Oversimplifying, this term refers to the developmental and evolutionary patterns of exchanges between living systems and their environment.

^4^Consider the extreme case of a creature with a totally different biochemical organization than ours (i.e., an alien). Because it is quite easy to imagine this living system having the same psychological disposition to feel pain as we do (for example), it is argued that psychology should deal with abstract generalizations rather than neural or physical substrates.

^5^This refers to the functional properties of “mirror neurons” – a set of sensorimotor neurons that fire when performing an action and when observing the same action performed by another individual (di Pellegrino et al., 1992; Gallese et al., 1996; Rizzolatti et al., 1996). Their activation, as Rizzolatti and Sinigaglia (2010) suggest, can transform the sensory representations of the others’ motor activity into a motor representation (a B-formatted representation) of the same action in the brain of the observer.

^6^Such insights have been systematically explored by scholars who defend a more radical view on ECS – ‘Enaction’ (see Di Paolo et al., 2017; Gallagher, 2017) – which puts major emphasis on how an organism’s cognitive complexity is recursively determined by (1) its own metabolic laws and (2) the (meta-metabolic) structures inherent to its niche (Maturana and Varela, 1980). For the present paper, however, we will mostly focus on the phenomenological roots of ECS.

^7^Pick-up games are traditionally defined as games that are spontaneously started by their participants.

^8^Consider how bodily based signals emerging from on-line action (e.g., the feeling of running too fast) can be associated with environmental affordances (e.g., the particular shape of the race track), and the ways in which they can inform the outcome of a given situation (e.g., a “wrong” feeling of take-off in a jumping the hurdle results in the athlete being more careful for landing). Because of the interplay of internal and external resources, they cannot be considered as B-formatted representations.

^9^For example, trampolinists in acrobatic sports reported a clear conscious awareness of what goes on in certain moments (e.g., Hauw and Durand, 2007).

^10^It emerged from the interviews that monitoring and control of the distance between swimmers are indeed their main concern.

^11^Trampoline jumping is characterized by a continuous act of compensation. As such, the main concern for jumpers is to agree on the degree of compensation required to help them maintain their timing.

^12^This aligns with recent work in sport science that emphasizes the role of auditory feedback in optimizing performance (e.g., using sounds to detect an opponent’s intention) (see Allerdissen et al., 2017; Camponogara et al., 2017; Sors et al., 2017, 2018).
